# Comparison of tomographic reports by radiologists and non-radiologists in trauma and interferences in management in a trauma reference center

**DOI:** 10.1590/0100-6991e-20233530-en

**Published:** 2023-06-22

**Authors:** GABRIEL MONDIN NOGUEIRA, LEONARDO KRIEGER RAFAEL, GABRIEL SEBBEN REICHARDT, MATEUS DALL’AGNOL, SILVANIA KLUG PIMENTEL

**Affiliations:** 1 - Universidade Federal do Paraná, Faculdade de Medicina - Curitiba - PR - Brasil; 2 - Universidade Federal do Paraná, Departamento de Cirurgia - Curitiba - PR - Brasil

**Keywords:** Tomography, X-Ray Computed, Diagnostic Errors, Multiple Trauma, Mental Fatigue, Medical Errors, Tomografia por Raios X, Erros de Diagnóstico, Traumatismo Múltiplo, Fadiga Mental, Erros Médicos

## Abstract

**Objective:**

diagnostic errors during the interpretation of an imaging test by the physician can lead to increased mortality and length of hospital stay for patients. The rate of divergence in the report given by a radiologist and an Emergency Physicians (EP) can reach over 20%. The objective of this study was to compare the unofficial tomographic reports issued by EP with the official reports issued by radiologists.

**Methods:**

a cross-sectional study, in which interpretations of the exams (documented in the medical records by the EP) of all patients undergoing computed tomography (CT) of the chest, abdomen or pelvis performed in the emergency room, at an interval of 8 months, were evaluated. These data were compared with the official reports of the radiologist (gold standard).

**Results:**

508 patients were included. The divergence between EP and the radiologist occurred in 27% of the cases. The most common type of divergence was the one not described by the EP, but described by the radiologist. The chance of having divergence in a case of multiple trauma is 4.93 times greater in relation to the case of only blunt trauma in one segment. A statistically relevant difference was also found in the length of stay of patients who had different interpretations of the CT scans.

**Conclusion:**

the study found a relatively high divergence rate between the EP report and the official radiologist report. However, less than 4% of these were considered to be clinically relevant, indicating the ability of the EP to interpret it satisfactorily.

## RESUMO

**Objetivo:**

os erros diagnósticos durante a interpretação de um exame de imagem pelo médico podem acarretar aumento da mortalidade e do tempo de internação dos pacientes. A taxa de divergência entre o laudo dado por um médico radiologista e a avaliação preliminar de um Médico Emergencista (ME) pode chegar a mais de 20%. O objetivo deste trabalho foi comparar as avaliações dos exames de imagem realizadas pelo ME com os laudos oficiais emitidos pelos radiologistas.

**Métodos:**

estudo seccional e transversal, no qual foram avaliadas interpretações dos exames (documentadas no prontuário pelos ME) de todos os pacientes submetidos à tomografia computadorizada (TC) de tórax, abdome ou pelve realizada na emergência, em um intervalo de 8 meses. Esses dados foram comparados com os laudos oficiais do médico radiologista (padrão ouro).

**Resultados:**

foram incluídos 508 pacientes no estudo. A divergência entre ME e radiologista ocorreu em 27% dos casos. O tipo de divergência mais incidente foi a não descrita pelo ME, mas descrita pelo radiologista. A chance de haver divergência em um caso de politrauma é 4,93 vezes maior em relação ao caso de somente trauma contuso em um segmento. Foi encontrada também uma diferença estatisticamente relevante no tempo de internamento dos pacientes que tiveram interpretações divergentes das tomografias.

**Conclusão:**

o estudo encontrou uma taxa de divergência relativamente alta entre o laudo do ME e o laudo oficial do radiologista. Contudo, menos de 4% dessas foram consideradas como clinicamente relevantes, indicando a capacidade dos ME em interpretar os exames de imagem de forma satisfatória.

## INTRODUCTION

The beginning of the study of radiological errors dates to 1899, with Beclere^1^, who analyzed the time it takes the retina to evaluate a fluoroscopic screen with adequate sensitivity. That study found that only after 20 minutes the retina has its maximum sensitivity, allowing the presence of interpretation errors before this interval, culminating in misdiagnosis. Diagnostic errors are a key component of medical error, representing up to 37% of that category^2^. In addition to causing an increase in mortality, diagnostic errors also increase patients’ length of stay, burdening health systems^3^. Medical malpractice moves millions of dollars annually in the judicial sphere and the focus of lawsuits is on emergency physicians (EP)^2^. This is due to the pressure exerted on this class and to the fatigue to which professionals are subjected to^4^. Most emergency rooms (ERs) do not have a radiologist readily available to interpret the requested imaging tests. Thus, in the urgency/emergency scenario, the EP initially interprets most exams, with the official report issued later by the radiologist. This is particularly important in trauma care services, as this type of care requires quick and assertive decisions. When analyzing 1,522 radiological exams, Mattsson and colleagues found a divergence rate of 20.35% between interpretations, with approximately one third of them being clinically significant^5^. Factors such as the patient’s age and studied anatomical region were risk predictors for the occurrence of discrepancies^5^. The occurrence of divergences also varies according to the type of exam. A 2019 study found a 6.5% rate of divergence between the interpretation of radiographs, indicating that the EP can interpret this exam with relative safety^6^. The type of sustained injury is also relevant when considering the number of discrepancies. A study conducted in South Africa analyzed only CT scans of polytrauma patients and found a concordance rate between the radiologist and the EP of only 58.6%^7^. However, only 4.84% of the patients were victims of a clinically relevant discrepancy, which was not statistically significant (p>0.05)^7^. The objective of this study was to compare the unofficial CT reports issued by the EP in the emergency department of a reference trauma center with the official reports issued by radiologists, analyzing their divergences and their clinical significance. We also analyzed the risk factors for the occurrence of such discrepancies.

## METHODS

This is a cross-sectional study conducted in a referral center for trauma care in the city and metropolitan region of Curitiba, State of Paraná, Brazil. Within an interval of eight months (10/01/2019-05/26/2020), we evaluated the medical records of all patients who underwent computed tomography (CT) of the chest and/or abdomen and/or pelvis performed in the emergency room. We reviewed the interpretations of the exams documented in the medical records by the EP (non-official report) and the conduct taken based on them. We then compared these data with the radiologists’ official reports, considered the gold standard. In cases where there was disagreement between the two interpretations, an experienced trauma surgeon analyzed the clinical significance of the disagreement independently. In cases where a sole case generated more than one discrepancy, we counted the discrepancy only once. We included patients over 18 years old (age at the time of collection) who underwent tomography of the chest and/or abdomen and/or pelvis in the period from 10/01/2019 to 05/26/2020. The exclusion criteria adopted were tomography performed for reasons unrelated to trauma, transcription of a verbal report provided by a radiologist simultaneously with the evaluation of the image by the EP, absence of an official CT report, lack of evaluation by the general surgery team, and return of the patient after previous trauma. In addition to the evaluation of the tomographic findings and their divergences, we analyzed patients’ gender and age, mechanism of trauma, type of trauma, time and date of the unofficial evaluation and date of the official evaluation, length of stay, and outcome. For a better evaluation, we divided the day into the following periods: morning (8h-12:59h); afternoon (1pm-5:59pm); night (20h-00:59h); and early morning (01h-05:59h). We also defined the periods close to the shift change in the morning (6:00 am to 7:59 am) and in the afternoon (6:00 pm to 7:59 pm). We considered divergences both the findings described by the radiologist and not by the EP and those that the EP described, and the radiologist did not. We divided the trauma mechanisms into categories, namely fall from the same level (FSL), fall from another level (FAL), stab wound (SW), gunshot wound (GSW), overturning, runover, car accidents, assaults, colliding with an object, hanging, crushing, motorcycle fall, horse fall, and bicycle fall. We reduced the variable type of trauma to blunt, open, or multiple trauma types. Polytrauma patients were those who suffered injuries in at least two different systems. When evaluating the official report, we considered only injuries related to the current trauma, disregarding old or clinical alterations. For age, we tested the null hypothesis that the age means are the same for non-divergent and divergent cases, versus the alternative hypothesis that the means are different. In addition, we tested the null hypothesis that the probabilities of death are the same for cases with and without divergence, versus the alternative hypothesis of different probabilities. For each of the categorical variables, we tested the null hypothesis that the probabilities of divergence between EP and Radiologist are the same for all classifications of the variable, versus the alternative hypothesis that the probabilities are different. We described quantitative variables by mean and standard deviation or by median, minimum, and maximum. We described categorical variables by frequency and percentage. To assess the association between demographic and clinical variables and the divergence between EP and Radiology, we fitted Logistic Regression models. We used the Wald test to analyze the significance of the variables. The estimated measure of association was the odds ratio, for which we computed 95% confidence intervals. We analyzed the association between divergence and death with the Fisher’s exact test. For the comparison between cases with and without divergence regarding length of stay, we used the non- parametric Mann-Whitney test. Values of p<0.05 indicated statistical significance. We analyzed the data using the Stata/SE v.14.1 software, StataCorpLP, USA. The study was approved by the Ethics Committee of Hospital do Trabalhador under number 32122820.8.0000.5225.

## RESULTS

WE analyzed 1,177 tomography records and, after applying the exclusion criteria, 508 patients formed the study group, of whom 78.1% (n=397) were male. The age of the patients ranged between 18 and 98 years, with a mean of 43.5±17.5 years. The most prevalent trauma mechanisms were automobile collisions (21.9%, n=128), falls from another level (15.9%, n=81), falls from the same level (12.8%, n=65), and the main type of trauma was blunt (68.9%, n=350). The most frequently performed imaging test was chest tomography (85.4%, n=434), followed by tomography of the abdomen (77%, n=391), and pelvis (75.8%, n=385). The most evaluated periods were night (28.4%, n=143), morning (20.5%, n=103), and dawn (19.5%, n=98). Divergence between the EP and the Radiologist occurred in 27% of cases (95% CI 23.1%-30.8%). The most frequent type of divergence was the one not described by the EP, but described by the Radiologist (59.9%, n=82), characterizing a false negative (Table 1). In this regard, the injuries that most caused false negatives were pulmonary contusion (30.5%, n=25), pleural effusion (28%, n=23), and rib fractures (20.7%, n=17).

**Table 1:** Type of divergence.

Divergence	n	%
Not described by EP / Described by Radiologist	82	59.9%
Described by EP / Not described by Radiologist	38	27.7%
Described by EP / Described by Radiologist*	17	12.4%
Total	137	100.0%

*Both professionals described different injuries.

[Table t2]

**Table 2 t2:** Type of divergence.

What the EP did not describe, and the Radiologist described	n	%*
Lung contusion	25	30.5%
Pleural effusion	23	28.0%
Rib fracture	17	20.7%
Hemothorax/pneumothorax	14	17.1%
Abdominal/pelvic hematoma	5	6.1%
Abdominal/pelvic free fluid	5	6.1%
Pneumoperitoneum	3	3.7%
Pericardial effusion	3	3.7%
Foreign body	2	2.4%
GIT hematoma	2	2.4%
Pneumomediastinum	1	1.2%
Liver injury	1	1.2%
Mediastinal shift	1	1.2%
Sternum fracture	1	1.2%
What the EP did not describe, and the Radiologist described	n	%*
Mediastinal hematoma	1	1.2%
Pancreatic injury	1	1.2%
Kidney injury	1	1.2%

*Percentages calculated on the total number of cases considered (n=82).

[Table t3]

**Table 3 t3:** 

What the EP described, and the Radiologist did not describe	n	%
Rib fracture	17	44.7%
Hemothorax/pneumothorax	5	13.2%
Liver injury	3	7.9%
Abdominal/pelvic free fluid	3	7.9%
Sternum fracture	2	5.3%
Pleural effusion	2	5.3%
Pericardial effusion	1	2.6%
Diaphragm elevation	1	2.6%
Splenic injury	1	2.6%
Lung contusion	1	2.6%
Pelvic fracture	1	2.6%
Abdominal/pelvic hematoma	1	2.6%
Diaphragmatic hernia	1	2.6%
Diaphragm injury	1	2.6%
Kidney injury	1	2.6%
Pneumoperitoneum	1	2.6%

*Percentages calculated on the total number of cases considered (n=38).

For the variables age, sex, and time of day, there was no significant association with the divergence between EP and Radiologist (Table 4). When comparing open and blunt trauma, we found no statistical significance for the occurrence of divergences (p=0.082). The comparison between polytrauma and blunt trauma showed statistical significance (p<0.001). The chance of divergence in a case of polytrauma was 4.93 times higher than the case of only blunt trauma in one segment (Table 4). There was also a longer hospital stay in cases of polytrauma. Also, we found no significant association between divergence and death.

**Table 4:** Trend factors.

Variable	Classification	n	% of cases with divergence	p*	OR	CI 95%
Age years)	(average ± pad dev)	Does not diverge: 42,9 ± 17,1 Diverg: 45,0 ± 18,6		0,249	1,01	0,99 - 1,02
Sex	Masculine	397	106 (26.7%)			
	Feminine	111	31 (27.9%)	0.797	1.06	0.66 - 1.70
Type of trauma	Blunt (ref)	350	66 (18.9%)			
	Open	55	16 (29.1%)	0.082	1.77	0.92 - 3.35
	polytrauma	103	55 (53.4%)	<0.001	4.93	3.08 - 7.89
Day period	Late change (ref)	56	14 (25.0%)			
(n=503)	Morning	103	27 (26.2%)	0.867	1.07	0.51 - 2.25
	Afternoon	94	25 (26.6%)	0.829	1.09	0.51 - 2.32
Variable	Classification	n	% of cases with divergence	p*	OR	CI 95%
	Night	143	38 (26.6%)	0.820	1.09	0.53 - 2.21
	dawn	98	28 (28.6%)	0.632	1.20	0.57 - 2.53
	change morning	9	4 (44.4%)	0.236	2.40	0.56 - 10.2

*Logistic Regression Model and Wald test, p<0.05.

We observed a statistically significant difference (p<0.001) in the length of stay of patients who had divergent interpretations of CT scans (mean of 6.6 days of hospitalization) when compared with those with concordant interpretations (mean of 1.1 days of hospitalization). These results are shown in Table 5. Of the 137 cases with disagreement, five (3.64%) were judged to be clinically significant. Table 6 brings their characteristics.

**Table 5:** 

Divergence	Hospitalization time (days)					p*
	n	Average	Median	Minimum	Maximum	
No	371	1.1	0	0	38	<0.001
Yes	137	6.6	2	0	120	

*Mann-Whitney non-parametric test, p<0.05.

**Tabela 6:** Description of cases with clinically significant divergence.

Variable	Case 1	Case 2	Case 3	Case 4	Case 5
Age at trauma (years)	20	29	33	40	45
Sex	Masculine	Masculine	Masculine	Masculine	Masculine
Trauma mechanism	GSW	SW	SW	Run over	Automobile collision
Type of trauma	Open	Open	Open	Blunt	Polytrauma
Day period	Afternoon	Dawn	Night	Dawn	Night
Death	No	No	No	No	Yes
Divergence	Yes	Yes	Yes	Yes	Yes
Described by EP	Yes	No	Yes	Yes	No
What the EP described	Hemothorax/	-	Pneumoperitônio	Contusão pulmonar, lesão renal hematoma abdômen/pelve	-
Pneumothorax	-	Pneumoperitoneum	Lung contusion, kidney injury abdomen/pelvis hematoma	-	Sim
Described by the Radiologist	Yes	Yes	No	Yes	Yes
What the Radiologist Described	Lung contusion	Pneumomediastinum and pleural effusion	-	Pneumothorax	AC fx, pulmonary contusion, hemothorax/pneumothorax, and abdominal/pelvis free liq
What theRadiologist Described	lung contusion	Pneumomediastinum and pleural effusion	-	pneumothorax	AC fx, pulmonary contusion, hemothorax/pneumothorax, and abdominal/pelvis free liq

*GSW: gunshot wound; SW: stab wound.*

## DISCUSSION

Radiology is an important ally during the diagnostic evaluation in trauma care. However, in developing countries like Brazil, there is not always a radiologist readily available in all trauma care services8. Issues such as fatigue and the complexity of the cases may be capable of increasing the incidence of divergences^4^ ^,^ ^9^. We analyzed the period of the day when the tomography was performed and the type of trauma in an unprecedented way. It is important to emphasize that the EP did not have formal training in Radiology, acquiring their experience during residency and throughout their careers. This study demonstrated that in terms of epidemiology, when analyzing the profile of patients undergoing tomography, there was a higher incidence of trauma in males, most of which were blunt and due to car collisions, which is consistent with the literature^10^ ^-^ ^12^. There was divergence between the EP assessment and the Radiologist’s report in 27% (n=137) of the cases, consistent with literature data^5^. When we analyzed their clinical relevance, the number dropped to just 0.98% (n=5). The most common type of divergence was the false negative, in which the Radiologist described the alteration in the exam and the EP did not. Although foreseen, documentation bias should also be considered. Contrary to what was expected, variables such as the patient’s age and time of day when the care was provided did not show a statistically significant increase in the divergence rate. As older patients have more associated diseases^13^ (distracting for those who analyze the tomography), we thought that would cause a greater number of discrepancies^5^. However, we did not observe this increase. The fact that the time of day did not change the divergence rate in a relevant way indicates that fatigue, in the specific conditions in which the on-call physicians were, was not a determining factor for the quality of interpretation of the image exams, which contradicts the data currently present in the literature^4^ ^,^ ^14^ ^,^ ^15^. Furthermore, there was no statistically significant association between the divergence rate and death, indicating that the types of divergence found were not relevant enough to change patients’ final outcomes.

When analyzing the type of trauma suffered, polytrauma, in addition to demonstrating a longer average length of stay, also determined a greater chance of occurrence of divergences (OR=4.93). In addition, there was a statistically significant relationship (p<0.001) between length of stay and the number of discrepancies found. However, we believe that the positive relationship between length of stay and the number of discrepancies found is because patients hospitalized for a longer time have a greater number of injuries, which increases the chance of them missing in the medical record (EP describing only the most important lesions). That is, the divergence found was not necessarily the causal agent of the longer hospital stay. In addition, physicians on duty, according to their experience, develop the ability to describe in the medical record only the injuries effectively capable of altering the prognosis of critically ill patients. Thus, many divergences found may be because the EP interpret the injury as insignificant, and not due to the lack of perception of the injury itself.

Of the 137 divergence cases found in the study, only five (0.98%) were clinically significant. Even though the data on divergences agree with the literature, the percentage of clinically significant divergences was significantly lower than another retrospective study (10.88%)^5^ and a prospective cohort (4.84%)^7^. Despite the occurrence of these cases, it is not possible to state that these divergences caused more morbidity and mortality, due to the retrospective nature of the study. The study has limitations, a retrospective bias^16^ and a documentation one, in which the EP may have described only the most important injuries, not documenting other findings. Likewise, there is the possibility of prior discussion about the case between the EP and the Radiologist, without reporting this in the medical record. Another limitation is that the study analyzed only CT scans of the thorax, abdomen, and pelvis, and did not consider other imaging modalities or even other evaluation sites, such as the head. The evaluation of only one trauma care center also limits this study.

## CONCLUSION

Although the study found a high rate of divergence between the EP reports and the Radiologists’ official reports, a small portion of these was clinically relevant. This demonstrates the attendants’ ability to satisfactorily interpret the images of the main life-threatening conditions in trauma.
